# Hypomethylating agents synergize with irinotecan to improve response to chemotherapy in colorectal cancer cells

**DOI:** 10.1371/journal.pone.0176139

**Published:** 2017-04-26

**Authors:** Anup Sharma, Rajita Vatapalli, Eihab Abdelfatah, K. Wyatt McMahon, Zachary Kerner, Angela A. Guzzetta, Jasvinder Singh, Cynthia Zahnow, Stephen B. Baylin, Sashidhar Yerram, Yue Hu, Nilofer Azad, Nita Ahuja

**Affiliations:** 1 Department of Surgery, The Johns Hopkins University School of Medicine, Baltimore, Maryland, United States of America; 2 Department of Oncology, The Johns Hopkins University School of Medicine, Baltimore, Maryland, United States of America; 3 Department of Gastrointestinal Surgery, Daping Hospital, Third Military Medical University, Chongqing, China; 4 Department of Urology, The Johns Hopkins University School of Medicine, Baltimore, Maryland, United States of America; University of South Alabama Mitchell Cancer Institute, UNITED STATES

## Abstract

Colorectal cancer (CRC) is the second leading cause of cancer death in the United States. In the metastatic setting, the majority of patients respond to initial therapies but eventually develop resistance and progress. In this study, we test the hypothesis that priming with epigenetic therapy sensitizes CRC cell lines, which were previously resistant to subsequent chemotherapeutic agents. When multiple CRC cell lines are first exposed to 500 nM of the DNA demethylating agent, 5-aza-cytidine (AZA) *in-vitro*, and the cells then established as *in-vivo* xenografts in untreated NOD-SCID mice; there is an enhanced response to cytotoxic chemotherapy with agents commonly used in CRC treatment. For irinotecan (IRI), growth diminished by 16–62 fold as assessed, by both proliferation (IC50) and anchorage independent cell growth soft agar assays. Treatment of resistant HCT116 cell line along with *in-vivo*, for CRC line xenografts, AZA plus IRI again exhibits this synergistic response with significant improvement in survival and tumor regression in the mice. Genome-wide expression correlates changes in pathways for cell adhesion and DNA repair with the above responses. A Phase 1/2 clinical trial testing this concept is already underway testing the clinical efficacy of this concept in IRI resistant, metastatic CRC (NCT01896856).

## Introduction

Colorectal cancer (CRC) is a common disease with a high incidence of cancer-related deaths in the United States and globally [1]. Over 50% of patients with CRC are eventually diagnosed with metastatic disease [2, 3]. Typically, there is a response to initial treatment with systemic chemotherapy, but over a period, tumors usually become resistant, spread and ultimately the patient succumbs to disease. Discovery of chemotherapy agents such as Oxaliplatin and Irinotecan have doubled survival in CRC patients from 11 months to 23–30 months but progress since has plateaued, even with newer targeted therapies [4, 5].

In designing strategies to improve the superior outcomes, epigenetic therapies are becoming an important potential strategy to augment efficacies of multiple treatment types [6, 7]. Changes in the epigenetic modulation of gene function are important determinants of cancer initiation and progression for CRC and other cancers [8]. Emerging evidence indicates that epigenetic abnormalities may also play a vital role in the development of chemoresistance in general and for CRC-specific agents including Oxaliplatin, Gemcitabine and Irinotecan (IRI) [6, 9–11]. Epigenetic therapy at low doses can cause reprogramming of the cancer cells as suggested by prior studies and such therapies may have the potential to reverse chemoresistance [12–15]

Commonly used hypomethylating agents (HMA’s) include nucleosides like 5-aza-cytidine (AZA) which inhibit DNA methyltransferase (DNMT) at low doses, leading to hypomethylation of DNA [16]. In the clinic, these inhibitors (DNMTi’s)—when combined other epigenetic modulators like histone deacetylase inhibitors (HDACi’s)—may yield enhanced effects, in part through augmenting re-expression of genes abnormally silenced in association with promoter hypermethylation. In this regard, there are signs in clinical trials in small subsets of patients employing epigenetic compounds for treating solid tumors (including lung and breast cancers) of improved overall survival [17–19]. Experience with these agents for treating hematologic neoplasms has primarily shown efficacy to the point that DNMTi's and HDACi's are FDA approved for myelodysplasia/acute myeloid leukemia (MDS/AML, and T-cell cutaneous lymphoma respectively [12, 20, 21]. One lesson learned from treating these following disorders is that the onset of the drug is slow and it may take several months for responses to occur [13]. Such time frames are particularly challenging for treating solid cancers in the setting of the significant burden of disease, and this has led to most strategies aiming at employing combinatorial epigenetic regimens [22, 23]. Previous studies by us and others have shown that even low dose treatment with a DNMTi such as AZA can reprogram cancer cells via activation of multiple cell signaling pathways that can allow the use of less toxic doses of chemotherapeutic agents [14, 24]. In this study, we test if epigenetic compounds can be combined with cytotoxic chemotherapy to improve the efficacy of chemotherapy, which could eventually improve outcomes in patients with metastatic CRC.

## Materials and methods

### Cancer cell lines and maintenance

The human primary colorectal cancer cell lines Caco-2 (ATCC^®^ HTB-37^™^), DLD-1 (ATCC^®^ CCL-221^™^), SW480 (ATCC^®^ CCL-228^™^), HT29 (ATCC^®^ HTB-38^™^), SW48 (ATCC^®^ CCL-231^™^), COLO 320HSR (ATCC^®^ CCL-220.1^™^), RKO (ATCC^®^ CRL-2577^™^), and Metastatic cell lines Lovo (ATCC^®^ CCL-229^™^), SNU-C1 (ATCC^®^ CRL-5972^™^), SKCO1 (ATCC^®^ HTB-39^™^), COLO 205 (ATCC^®^ CCL-222^™^), COLO 201 (ATCC^®^ CCL-224^™^), SW620 (ATCC^®^ CCL-227^™^), T84 (ATCC^®^ CCL-248^™^) were obtained from the American Type Culture Collection (ATCC, Manassas, VA, USA). ATCC recommended conditions be used to maintain the cell lines.

### MTT cell viability assay

Following treatment with AZA for 72 hours, cells were seeded in triplicate in a flat bottom 96 well plate for the cell viability. Cells were treated with different concentrations of IRI, for 48 hours. Cell viability was assessed using the CellTiter 96^®^ AQueous Non-Radioactive Cell Proliferation Assay (MTS) (Promega) and quantified on a SpectraMax M2E plate reader (Molecular Devices). Following this, IC50 of colorectal cancer cell lines was determined (S1 Fig). Raw data were corrected for background absorbance, and these data points were used to establish IC50 drug dose (drug dose causing 50% growth inhibition). GraphPad Prism non-linear (curve fit) regression algorithms were used to calculate the drug dose causing 50% growth inhibition (IC50 drug dose). Experiments were repeated twice to ensure reproducibility. Data points were plotted plus or minus standard deviation (SD). Assessment of Synergy was assessed using Chou-Talalay’s plot in Compusyn software [25]**.**

### Methylation of the LINE-1 promoter

PCR and pyrosequencing for LINE-1 methylation were performed as previously described [26]. Briefly, PCR followed by pyrosequencing using the PyroMark kit (Qiagen, Valencia, CA). The relative amounts of C in the 3 CpG sites was used as overall LINE-1 methylation level in cell lines examined. The experiment was conducted twice to ensure reproducibility. We have plotted mean values for representation of global demethylation for each cell lines.

### Western blot analysis

Western blot analyses were performed as follows. First, The baseline levels of DNMT1 in different CRC cells was determined on untreated cell lines and normalised to the β-Actin levels. This was achieved by plating cells at a confluence of 50–70% after 48 hrs of plating in the growth medium. Further, colorectal cells were treated with specified concentration of AZA as described earlier. Treatments were carried out for 72 hrs with the addition of new AZA, as determined earlier [27]. Post treatment, total protein was isolated, and ten micrograms were used for electrophoresis and blotted onto PVDF membrane. Primary antibodies diluted in blocking buffer (5% milk as per antibody specifications) to a 1:1000 dilution. Secondary antibodies for housekeeping proteins such as actin, used as internal controls, were diluted at 1:2000. Blots were developed using ECL (GE Healthcare). Primary and secondary antibodies purchased from Cell Signaling.

### Tumor xenograft assay

NOD-SCID mice were purchased from The Jackson Laboratory (Bar Harbor, ME, USA), and cared for in strict accordance with an approved Johns Hopkins IACUC protocol. Colon cancer cells were pre-treated with 500 nM AZA or PBS (Mock) for 72 hours followed by another seven days in culture without the drug. Harvested cells were injected (1×10^6^) subcutaneously with 50% Matrigel basement membrane (BD Biosciences, Billerica, MA) into both flanks of 4 to 6 week-old NOD/SCID mice. Tumors were measured weekly and volume calculated as LxWxH (mm^3^). Protocols for all animal experiments conducted at Johns Hopkins were approved by the John Hopkins University Animal Care and Use Committee guidelines [28].

### Soft agar assay

Colorectal cancer cells were plated in either six well plate or 60 mm dishes and treated with 500 nM of AZA for 72 hours followed by IRI treatment for 48 hrs. Following treatment, 4000 cells from treated and control group were plated in triplicate in 60 mm dishes and incubated further for seven days. Crystal violet (Sigma-Aldrich) solution was used to stain colonies. For HCT116, the dose selected for short term treatment was 10 nM and had to be reduced to 5 nM for long term treatment due to pronounced cytotoxicity on treatment with IRI. For the long term treatment in HCT116 cells were pretreated with 500 nM of AZA for 48 hours while cells were in log growth phase, media renewed every 24 hours. Media was replaced at the end of 48 hours and cells were maintained in log growth phase for five days. This schedule of AZA treatment for 48 hours and subsequent rest period for five days was repeated three more times. On day 28, cells were split, counted and re-plated in the 6-well plate for the next steps. After the epigenetic treatment, cells were then treated with different concentration of IRI for 48 hrs, and media was replaced. Cells were allowed to grow for 7–10 days. Colonies were then fixed and stained with using crystal violet.

### Therapeutic administration of IRI following AZA treatment

NOD-SCID mice were purchased from The Jackson Laboratory (Bar Harbor, ME, USA), and cared for in strict accordance with an approved Johns Hopkins IACUC protocol. NOD-SCID mice aged 4–6 weeks were injected subcutaneously with 1 million colon cancer cells with 50% Matrigel basement membrane (BD Biosciences, Billerica, MA) in each flank. The mice were randomized into four different treatment arms: Control treatment, AZA treatment alone, IRI treatment alone, AZA and Irinotecan (AZA+IRI) treatment combination. Three days after cell injections, mice in the AZA and the combination arm received daily i. p. injections of AZA (Sigma-Aldrich, MO; 1 mg/kg) diluted in sterile saline for five days followed by a two-day rest period. The ‘control’ mice and IRI mice received saline instead of AZA. All tumors were measured bi-weekly and volume was calculated as H×L×W (mm^3^). When tumors reached a volume of 500 mm^3^, IRI and AZA+IRI mice received biweekly i. p. injections of IRI (Sigma-Aldrich, MO; 50 mg/kg) diluted in sterile saline. Treatment continued (except if indicated otherwise) until tumors reached maximum allowable size 2000 mm^3^ (used as an endpoint for survival studies). Mean tumor growth inhibition was calculated as TGI = (1-(Tf-T0)/(Cf-C0)) *100, where Tf and T0 represent final, and original mean tumor volumes in the treatment arm, respectively, and Cf and C0 represent definitive and initial mean tumor volumes in the vehicle control arm, respectively.

### Expression arrays

Following the treatment with 500 nM of AZA as mentioned above, cells (Caco-2 and SW480) were then treated with IRI or control, at a concentration corresponding to the IC50 on *in- vitro* assays. Cells were flash frozen after 36 hours in IRI media. RNA was extracted using standard protocol [14] hybridized to Agilent Human 4×44K expression arrays (Agilent Technologies, Santa Clara, CA, USA. Feature extracted values files were imported into R using the read.maimages function available through Limma [29]. Arrays were subsequently [30] subjected to within-array normalization (loess), and between-array normalization (Aquantile) [31], which resulted in high-quality normalization across all nine arrays (S2 Fig).

To identify differentially-expressed probes, The treatment factor was created across all 18 channels and the effect of the treatments was used to model [32] normalized values using the lmscFit function [33, 34] from limma across all channels, and those probes that—following Benjamini-Hochberg multiple testing adjustment had p-values of <0.05 in the contrast of interest were considered differentially expressed. The “Sensitization Genes” were created by generating the differentially expressed probe list from the AZA+IRI-Mock contrast, as well as the IRI-Mock contrast, and the Gene IDs from the AZA+IRI-Mock differentially expressed probes list that was not present in the IRI-Mock contrast were called the Sensitization Genes. This list of Sensitization genes is available in S1 Table. The Expression matrix for the Sensitivity genes was generated by taking the mean of the log fold change for each comparison of interest for each Gene ID from S1 Table. This expression matrix is available in S2 Table.

## Results

### HMAs are effective at low doses in causing global demethylation in CRC cell lines

**Fig 1:**
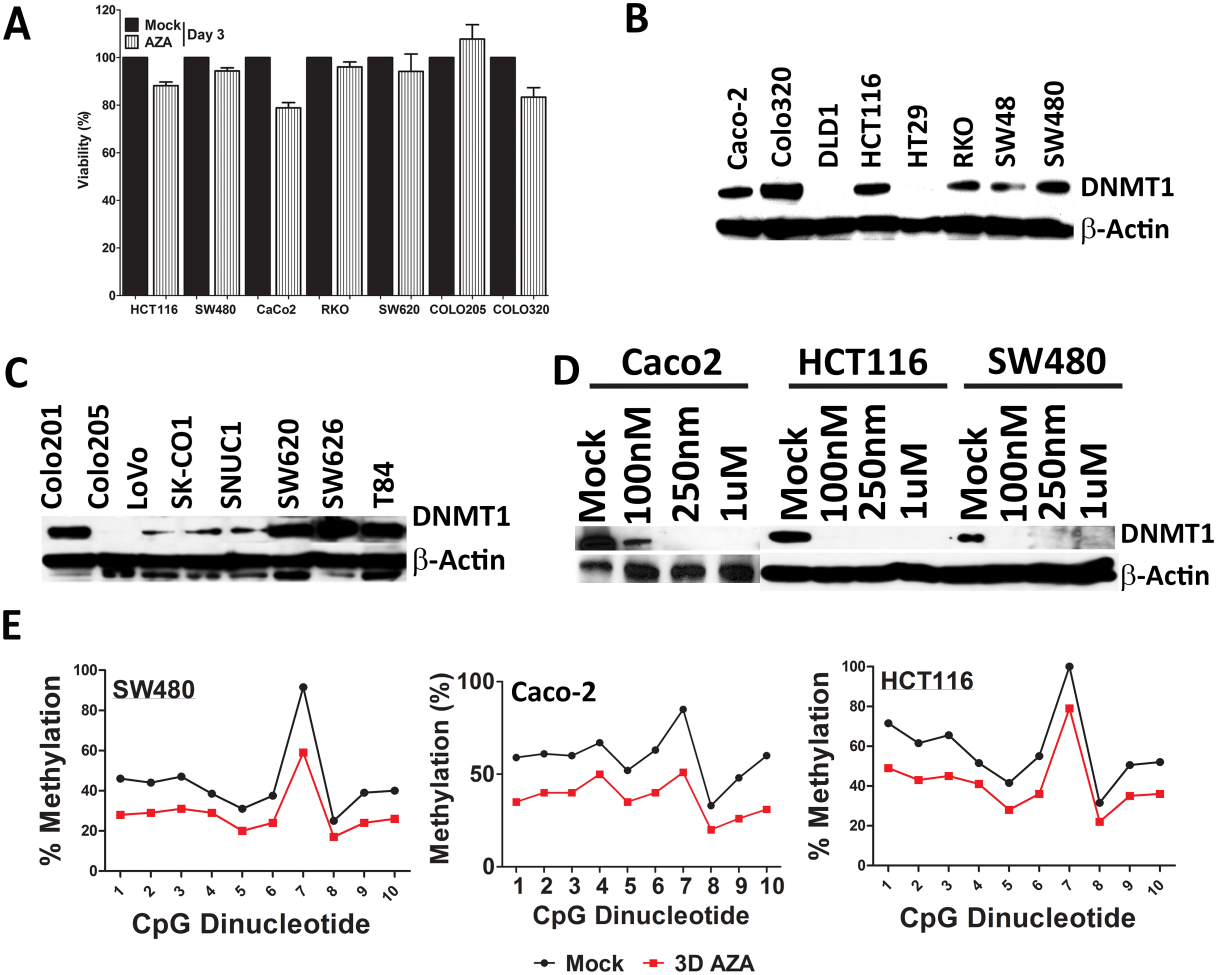
Low dose HMA causes global demethylation in CRC cell lines. **(A)** Colon cancer cell lines (HCT116, SW480, Caco-2, RKO, SW620, Colo205, Colo320) showed very little or no cytotoxicity. Percent viability after 72 hrs treatment with 500 nM AZA, relative to mock control. Bars represent the mean of 3 replicates ±SD; **(B & C)** Colon Cancer cell lines saw basal or low expression of DNMT1 protein. Upper and the lower panel is a representative blot for DNMT1 and β-Actin as a housekeeper. **(D)** Protein expression of DNMT1 in colon cancer cells (Caco-2-, HCT116 and SW480) changes after treatment with a various concentration of AZA. Cells were treated with AZA at the indicated concentrations for 72hr and then subjected to immunoblot analysis using anti-DNMT1 antibody. Actin served as a loading control. B, C, and D are representative blots of at least three independent experiments. **(E)** Quantitation of DNA methylation using bisulfite LINE-1 PCR and Pyrosequencing in Caco-2, SW480 and HCT116 (3-day treatment followed by 3 rest period). Representative LINE-1 quantitation presented for the cell lines. The pyrogram quantitates C for methylated, and T for unmethylated DNA was plotted as a line graph.

When low doses of AZA were used as a treatment for various CRC cell lines, only modest (3.9 to 18%) initial cytotoxicity is observed (Fig 1A). The basal expression levels of DNMT1 in different CRC cells was variable: in some cases cells lines such as Caco-2, DLD1, HT29, Colo205, LoVo, SKCO1, and SNUC1 show very low or no basal levels, while others such as Colo320, HCT116, SW620, SW626, and SW480 showed higher levels (Fig 1B and 1C). The downregulation of DNMT1 by varying doses of AZA in HCT116 and SW480 showed depletion of DNMT1 (Fig 1D) even at low levels, except Caco-2 which showed decreased but not complete inhibition at low (100 nm) concentration (Fig 1D). We further tested global methylation levels after treatment with AZA (72 hrs, followed by 3 days of rest period) in multiple CRC cell lines at a low dose (500 nM) by measuring methylation levels of LINE-1 retrotransposons at various time points [35]. The AZA doses also cause demethylation of LINE-1 retrotransposons at different time points [35] showing global demethylation (Fig 1E).

### Low doses of AZA have profound effect on tumor burden

**Fig 2:**
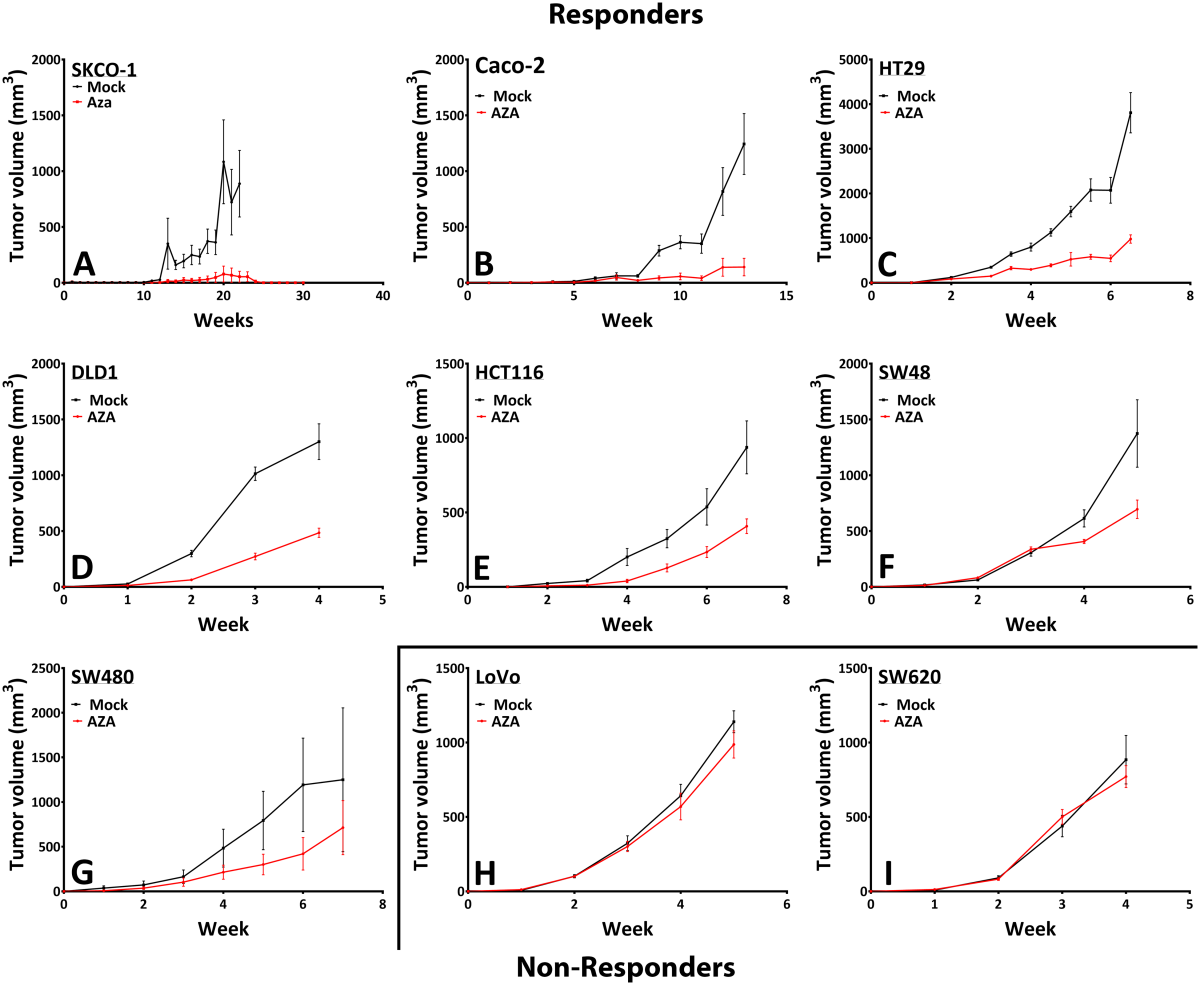
*In vivo* epigenetic therapy sensitize CRC cell line xenografts to decrease tumor burden. Colorectal cancer cells were treated for 72 hours with AZA at 500nM and saline, or PBS inject Mock, were allowed to recover from the short-term cytotoxic effects of AZA for seven days before injection into NOD-SCID mice (n = 10 in each case). NOD-SCID mice were xenografted with various CRC cell line and monitored until mice showed 2000 mm^3^ tumor development. **(A-G)** Represents xenografted mice responded to therapy and **(H & I)** are the mice which did not respond to the treatment **(**S3 Fig**)**. Mean tumor volume (±SEM) over time are plotted. Statistical significance determined by two-tailed paired t-test.

In *in-vivo* studies, we have previously demonstrated that short-term exposure (72 hr) with low doses of AZA to cultured CRC cells has a profound memory effect wherein these are severely blunted for growth over multiple passages when implanted into immunosuppressed mice [27]. Similar results are now seen when such studies are expanded to a panel of 15 CRC cell lines. Seven of these (SK-CO1, Caco-2, SW480, DLD1, SW48, HCT116, and HT29) out of 15 showed an overall decrease in tumor burden (greater than 25% decrease in tumor burden, P<0.002), (Fig 2A–2I & S3A–S3F Fig).

### Epigenetic therapy with HMA sensitizes to chemotherapy

**Fig 3:**
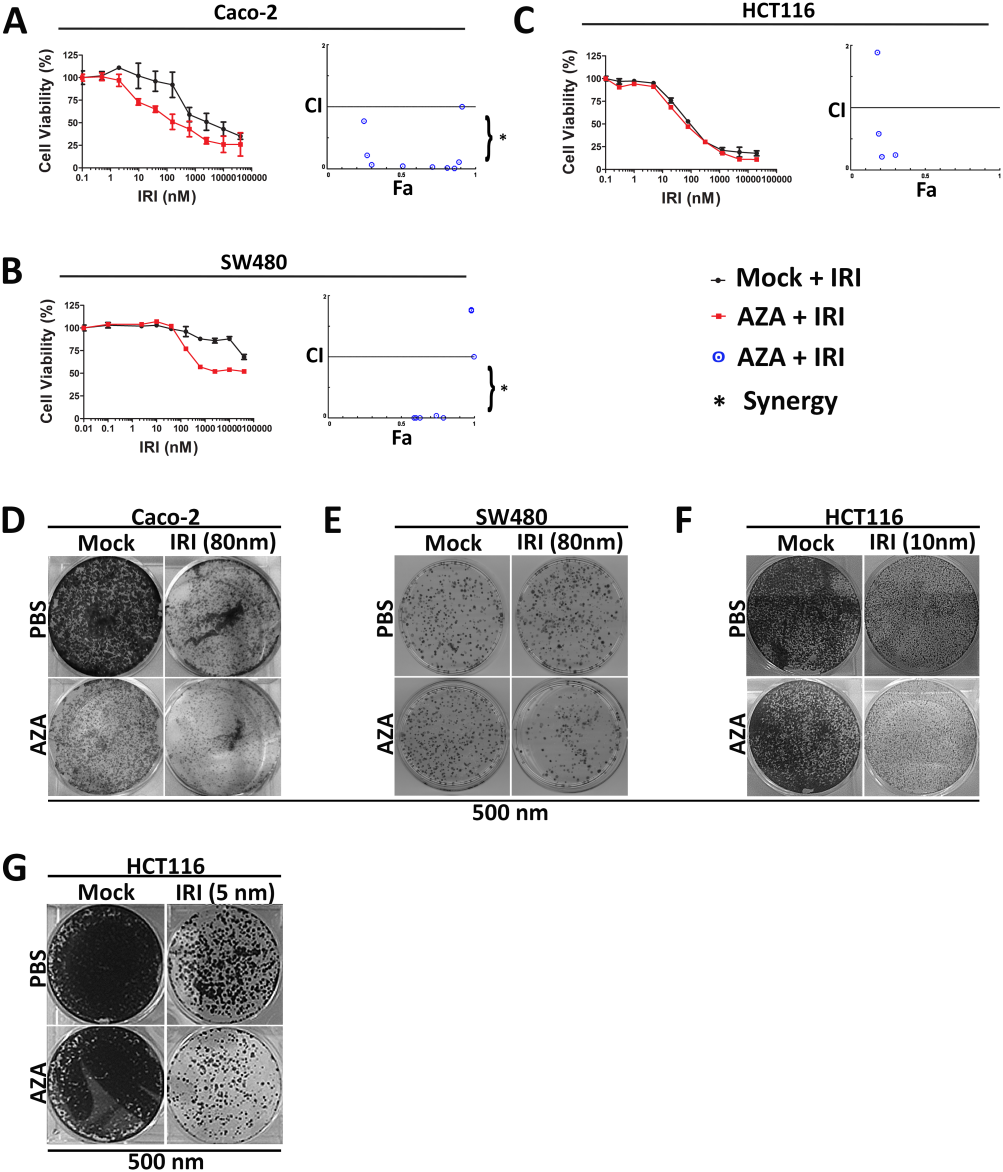
Epigenetic therapy sensitizes chemotherapy in CRC cell lines. **(A-C)** Log dose response curves for CRC cell lines treated (in triplicate) with IRI for 72 hours three days’ post epigenetic therapy. Individual curves represent the percentage of viable cells (±SD) for each epigenetic pretreatment condition normalized to its untreated control cells, such that the highest values for each pretreatment state represent 100%, and 0%. Data shown from representative experiments. Visualization of drug interaction is illustrated by CI-Fa plot. The CI-Fa plot represents the combination index plotted versus Fa, the fraction of affected enzyme or biological function. CI values were calculated from each Fa (i.e. various drug concentration) for CRC cell lines Caco-2, SW480, and HCT116. Average synergism (CI<1) at Fa>0.5 for all three CRC lines. The AZA and IRI chemotherapeutic doses may be significantly reduced for combinations that are synergistic at Fa>0.5 for all three cell lines. **(D-F)** Caco-2, SW480, and HCT116 cells were seeded on a solidified Matrigel layer six days after epigenetic therapy. Beginning the following day, cells were treated with chemotherapy for 72 hrs. The drug was then removed, and colonies were permitted to grow 2–4 additional days. Representative Caco-2, SW480 and HCT116 colonies following treatment with 80 nM IRI. Three independent experiments (total nine replicates. **(G)** HCT116 colony forming assay examined for prolonged exposure to AZA as a pretreatment to IRI on HCT116 cells. HCT116 cells were pretreated with AZA for 72 hours, followed by five days’ rest and the schedule was repeated three more times before IRI treatment in soft agar assay. This modification increased colony growth inhibition in HCT116 significantly more than IRI alone, which were previously resistant.

We explored the possibility that AZA (500 nM) would make sub-lethally treated CRC cells more susceptible to chemotherapy. The above pretreatment paradigms can add differentially to the effects of chemotherapeutic drugs commonly used in the treatment of CRC such as irinotecan, oxaliplatin, etc. Of the various compounds tested (Oxaliplatin, Cisplatin, Irinotecan, data not shown), only Irinotecan showed synergy with AZA. CRC cell lines (Caco-2 and SW480) exhibited dose-dependent responsiveness to HMA and chemotherapy drugs IRI (Fig 3A & 3B). Exposing cells to IRI alone resulted in higher IC50 (Caco-2 IC50 was 1.25 μM, SW480 was 20 μm and HCT116 was 10 μm) for CRC cells. In contrast chemo priming with AZA lowered IC50 to 78 nM (compared to 1250 nM; a ~16-fold improvement; P<0.0025 in Caco-2), and to 312 nM (compared to 2000 nm, >62.2-fold increase; P<0.04 in SW480) on pre-treatment followed by resting (S1 Fig). However, there was no improvement for HCT116 with AZA pretreatment (Fig 3A–3C). The combination of both AZA and IRI showed dose-dependent growth inhibition in Caco-2 and SW480 and enhanced lethality response to IRI treatment for Caco-2 and SW480 cells (Fig 3A & 3B), however, it failed to inhibit growth in HCT116 cells (Fig 3C). The resulting combination indices (CI) theorem of Chou-Talalay for additive effect (CI = 1), synergism (CI < 1), and antagonism (CI > 1) revealed the combination of both drugs to have synergistic improvement in responses to IRI in both Caco-2 and SW480, but not in case of HCT116 (Fig 3A–3C).

AZA priming followed by IRI inhibition also resulted in an enhanced reduction of cellular soft agar cloning for both Caco-2 and SW480 cell lines when IC50 doses of AZA + IRI was used for treatment (Fig 3D–3F). However, once again HCT116 cells did not show any significant decreases in colony formation (Fig 3F). However, when HCT116 cells were pretreated for a prolonged period (AZA for 72 hours followed by five days' rest and repeating this schedule 3 more times before IRI treatment), we observed growth inhibition of the previously resistant cells (Fig 3F–3G). Sensitivity to IRI was established based on clinically available concentrations.

### Epigenetic priming followed by irinotecan decreases tumor burden in mice models

**Fig 4:**
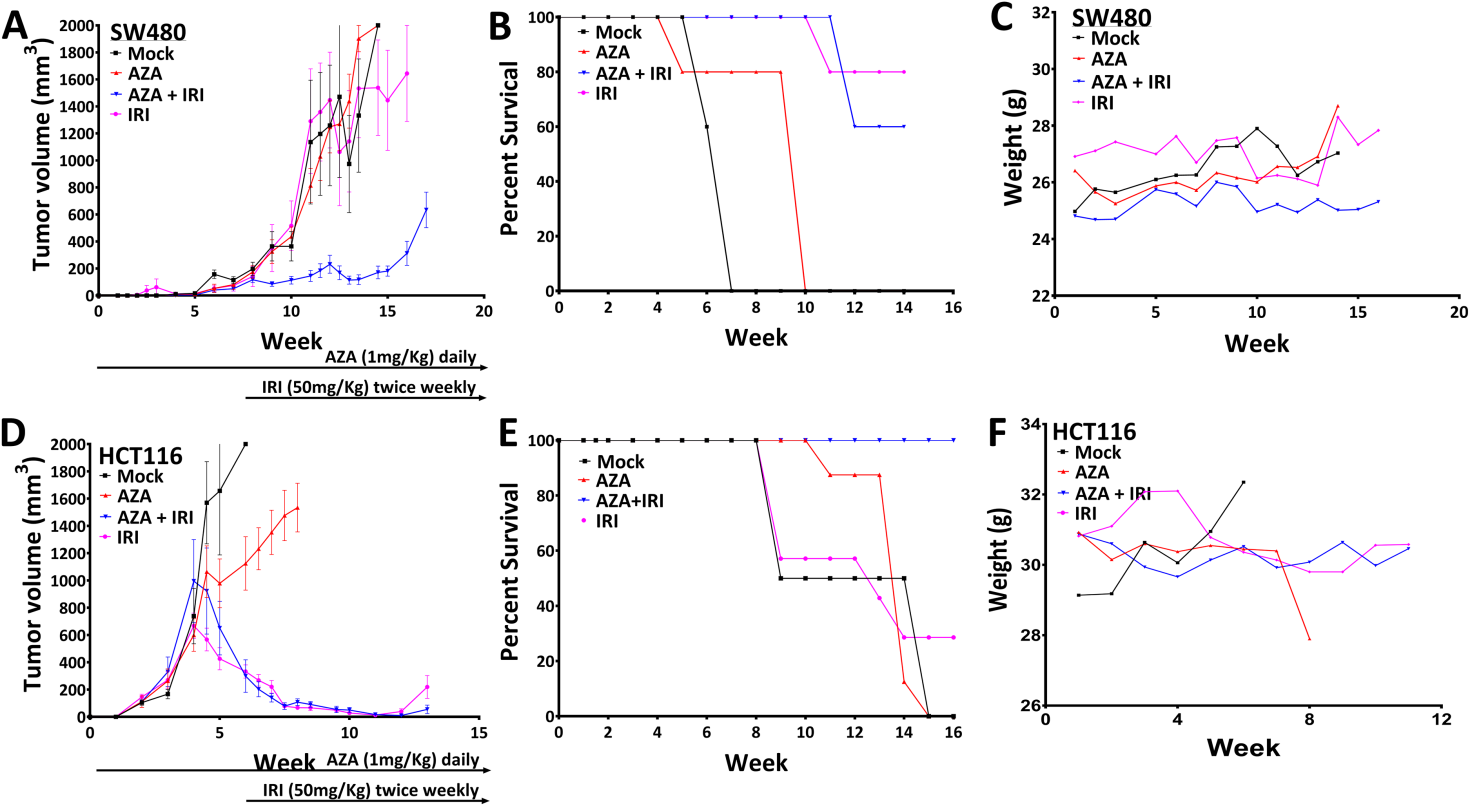
Epigenetic therapy *in vivo* sensitize SW480 xenografts to subsequent chemotherapy. NOD-SCID mice bearing SW480 and HCT116 xenografts were treated with 0.5 mg/kg AZA (sc, qd × 5) and 10 mg/kg IRI (i.p., day 5), or vehicle, until the end of experiment cycles. **(A&D)** Mean tumor volume (±SEM) over time. Statistical significance determined by two-tailed paired t-test. **(B&E)** Survival analysis showed that the median survival for SW480 and HCT116 tumor-bearing mice was significantly reduced in AZA and IRI treated compared to mice which were untreated tumors. **(C&F)** Indicates weight changes over the course of treatment. Mice were either treated with AZA alone, AZA + IRI or IRI alone or saline vehicle. Mice were treated until the end of the experiment. N = 10 mice in each.

The above epigenetic priming in combination with IRI *in-vitro* also is effective in xenograft tumor responses in immune-deficient mice. Intraperitoneal administration of AZA (1 mg/kg) and IRI (50 mg/kg) alone, were all well tolerated as demonstrated by animal weights. While each drug alone showed no or minimal decrease (5%) in tumor growth (Fig 4A), AZA and IRI in combination produced a profound decrease in tumor xenograft growth (90% reduction in growth) (P<0.05). Moreover, survival was dramatically improved with combination drug administration (>12 weeks’ vs. seven weeks for the mock, P<0.0321) (Fig 4B), and the combination treatment was again well tolerated by the mice (Fig 4C). For xenografted HCT116 cells, IRI alone reduced tumors by 80% (Fig 4D) and, thus the effects of synergy with AZA were not observed (Fig 4E). However overall survival was again improved (>16 weeks' vs. 12 weeks in mock (P = 0.0056) without any mouse toxicity (Fig 4F).

### Gene expression profiling of responder cells to combination treatment

**Fig 5:**
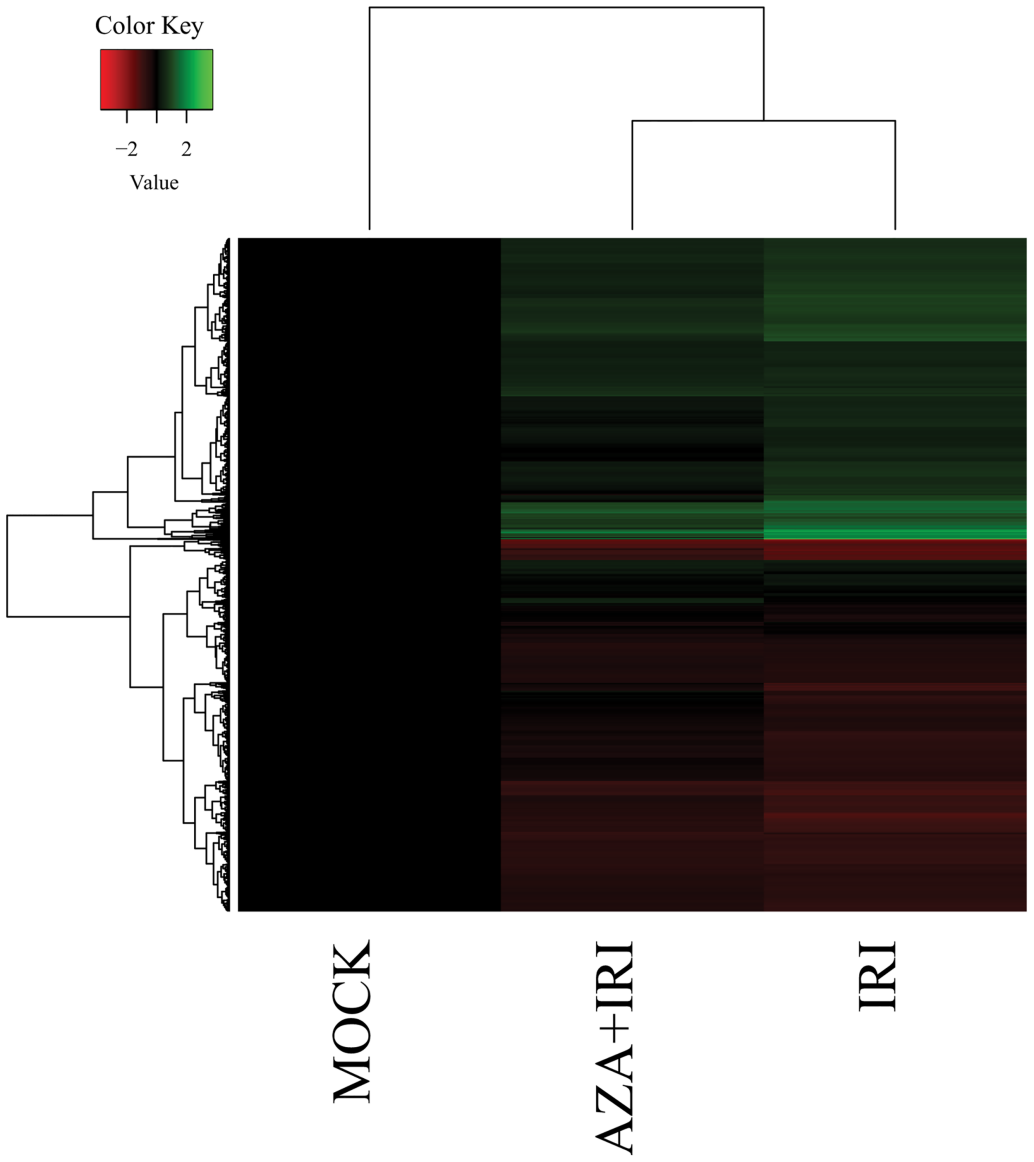
Relative expression of Caco-2 and SW480 colon cancer cell lines. Heatmap showing relative expression (Mock is shown as reference) of the Sensitization Genes in the IRI and AZA+IRI-treated samples.

Since AZA treatment leads to changes in gene expression and these changes could play a role in the sensitization of CRC cell lines to IRI, we performed gene expression profiling on cells following the combination treatment (Fig 5). One thousand four hundred fifty-three genes showed altered gene expression after the above treatments of cultured cells and we classified these as potential “Sensitization genes.” The biological process terms that were enriched (following a Benjamini-Hochberg multiple testing adjustment) were cell-cell adhesion (GO:0098609: genes included: CHMP5, S100B, among others), regulation of transcription, DNA-templated (genes involved: MED23, HOXD3, and ATRX—among others) and DNA repair (genes included: FANCA1, FEN1, and MSH3 –among others). We also observed differential expression of three different ABC transporter genes (ABCD1, ABCA4, and ABCC1). ABC transporters are often involved in the extracellular transport of drugs. Interestingly, ABCC1 was downregulated approximately 1.5 fold in the pretreated cells relative to the non-pretreated cells. The complete list of enriched GO terms is provided in S2 Table.

## Discussion

Despite multiple approved therapeutic options, long-term prognosis remains dismal for CRC patients with progressed disease. An epigenetic approach to reversing CRC chemoresistance may pave the way for innovative therapeutic strategies and improved outcomes, as emerging evidence both from our laboratory and others’ indicates that epigenetics may play a major role in the development of chemoresistance [23, 36]. Our preclinical data suggests that therapy with IRI could potentially be used in conjunction with HMA’s in CRC for better efficacy of the chemotherapeutic drug.

Our current study shows treatment with the low dose HMA's can cause profound global demethylation changes (Fig 1E) over a prolonged period. This not only achieves gene expression changes critical to tumorigenesis, but these changes occur without any noticeable cytotoxic effects (Fig 1A). In this preclinical study, exposure of HMA’s followed by a week of rest showed “memory” like effect causing significant tumor regression. This result may resemble the clinical response seen in patients with hematological malignancies [13] and other solid tumors like Non-Small Cell Lung Cancer (NSCLC) [36]. Tumor regression in xenograft model was significant and with a degree of sensitivity (Fig 2A–2I) (over a period of 10 to 30 weeks), highlighting different genetic makeup of the tumor development. Due to this, some were non-responsive, displaying delayed regression, while the remaining showed an immediate response. All the above indicates that HMA compounds may display memory effect, which is pronounced once the cytotoxic pressure of the drug is weaned off from the circulation.

Importantly, we have now tried to highlight the fact that low dose HMA treatment causes sustained gene expression changes which could contribute to the emergence of a new phenotype. This leads to tumor regression caused by modulation of multiple key pathways, such as apoptosis, DNA repair and down-regulation of the cell-cell adhesion and others, which persist once drug pressure is removed causing synthetic lethality leading to cell death. This memory-like effect indicates that the tumor cell might be undergoing some reprogramming that could be a desirable effect and potential therapeutic application.

Importantly, and the key to our findings, a major function of DNA methylation in CRC cell lines is the silencing of gene expression pathway active during oncogenesis [37]. An interesting observation was the selective response of HMA to cell lines Caco-2 and SW480 while no response seen in HCT116 CRC cells in-vitro. This non-responsive cell line showed a response to prolonged treatment with HMA followed by chemotherapy. HCT116 which was previously non-responsive is now responsive to the extended treatment, indicating that more time is required for an effective response. One interpretation of this observation is that cells need more time to recover from the cytotoxic pressure of the drug before it can synergize with the drug to slow down the proliferative process of the tumor cell. Our treatment protocol could allow the cell to incorporate AZA, which binds on the dividing DNA strand which on division is magnified to cause inhibition on treatment with IRI, which acts on the DNA polymerase activity. This difference in response could be due to the hypermethylated state of DNA in HCT116 cells which are resistant to the treatment, suppressing the expression of tumor suppressor genes, which control many cellular functions, including proliferation. Following treatment with the HMA, these genes are re-expressed in resistant HCT116 cells, and hence the effect is more pronounced.

These results are promising and, suggest a clinically relevant shift in chemotherapy responsiveness. Intriguingly enough, the continued treatment with HMA may also allow synergy to manifest, as seen by the data in HCT116, where short term treatment on soft agar doesn’t show response but continued treatment shows a significant decrease in colony growth on soft agar assays. Extrapolation of this data to patients would suggest that some patients may respond quickly to HMA, but others need time to respond to such therapies, such that trial design endpoints may need to be redefined to allow alternative endpoints.

Finally, in addition to addressing the issue of efficacy in vivo without causing toxicity, both SW480 and HCT116 xenograft model show remarkable tumor regression with prolonged survival in comparison to the control mice in the presence of combined subsequent treatment of both drugs. However, the HCT116 xenograft on treatment in mice, showed remarkable tumor regression and survival, although indicating no synergy between the drugs used for treatment. This could be related to the rest cycle after AZA treatment, allowing cells to harmonize the oncogenesis and tumor suppressor pathway which enhances survival in comparison to the IRI treatment alone.

We also examined gene expression changes to understand the mechanism of responses. We found that genes involved in cell adhesion and DNA repair were differentially expressed in the mock-AZAIRI comparison, but not in the mock-IRI comparison. This suggests that AZA-preconditioned cells are deregulating these pathways, and this may play a role in increased IRI chemosensitivity. Various aspects of what is broadly referred to as “cell adhesion” have previously been implicated in both metastasis and chemosensitivity [38–40], and a role for DNA double-strand breaks are well-established in IRI sensitivity [41]. These observations give support to our overall hypothesis that epigenetic marks regulate genes essential to chemosensitivity. We also observed the dysregulation of a number of ABC transporter genes. Of particular interest is the approximate 1.5-fold downregulation of the IRI efflux transporter ABCC1 in the preconditioned cells relative to the untreated, which would suggest that at least part of the resistance could be the consequence of poor removal of cellular IRI. We anticipate this will be a fruitful avenue for future insights into how cancer cells become resistant to treatments may lead to the development of novel therapies.

In this study, we show that AZA can synergize with IRI using both *in-vitro* and *in-vivo* models in colorectal cancer cell lines. Based on the promising study results, we have initiated a Phase1/2 study investigating if treatment with an HMA can reverse chemoresistance to irinotecan in metastatic colorectal cancer cell patients that have developed resistance to IRI. We used a novel HMA, guadecitabine that has been shown to be clinically and biologically active in patients with myelodysplastic syndrome and acute myeloid leukemia [26]. The Phase 1 study testing the combinatorial drug dosing and the schedule has recently been completed and results were reported [42]. A Phase 2 randomized, international clinical trial testing this concept is now underway, testing the clinical efficacy of this concept in irinotecan-resistant, metastatic CRC (NCT01896856). This approach, if successful, may have broad applicability in personalized treatment.

## Supporting information

S1 Fig IC50 of the three colon cancer cell lines. Three cell lines Caco-2, SW480, and HCT116 IC50 was determined.
(TIF)

S2 Fig Boxplot of M values. A log2 ratios between red and green signals in all nine arrays from this analysis following full normalization procedure (see Materials and methods for details).
(TIF)

S3 Fig *In-vivo* epigenetic therapy sensitize CRC cell line xenografts to decrease tumor burden. **(A-F)** Represents non-responder xenografted mice to the treatment. Mean tumor volume (±SEM) over time are plotted. Statistical significance determined by two-tailed paired t-test.
(TIF)

S1 Table List of sensitization genes. Official Gene Symbols of the Sensitization genes, derived as described in Method and Materials.
(XLSX)

S2 Table Expression matrix of sensitivity genes. Derived as described in Methods and Materials. All values are log_2_ transformed.
(XLSX)
