# Correction: Hypomethylating agents synergize with irinotecan to improve response to chemotherapy in colorectal cancer cells

**DOI:** 10.1371/journal.pone.0242750

**Published:** 2020-11-30

**Authors:** Anup Sharma, Rajita Vatapalli, Eihab Abdelfatah, K. Wyatt McMahon, Zachary Kerner, Angela A. Guzzetta, Jasvinder Singh, Cynthia Zahnow, Stephen B. Baylin, Sashidhar Yerram, Yue Hu, Nilofer Azad, Nita Ahuja

The following information is missing from the Funding statement: This study was funded by a grant to Dr. Ahuja from Astex Inc. (GRANT # 120039).

There is information missing from the Competing Interests statement. The correct Competing Interests statement is as follows: Dr. Ahuja receives research grant funding from Astex Inc. and the Van Andel Research Institute. She is a consultant for and has licensed methylation biomarkers to Cepheid (PATENT # 10167513). Dr. Ahuja has also served as consultant to Johnson and Johnson, an advisor to Celgene, and a member of the Scientific Advisory Council to the No Stomach for Cancer Foundation. This does not alter our adherence to PLOS ONE policies on sharing data and materials.

The raw data underlying the findings of this study are missing from the list of Supporting Information. The authors have provided the data as Supporting Information file S1 Data. With this correction [1], all relevant data are now provided.

## Supporting information

S1 Data Chemopriming data. (XLSX)
