# Supplementary figures and images for: Hypomethylating agents synergize with irinotecan to improve response to chemotherapy in colorectal cancer cells
Source: PLoS One. 2017 Apr 26;12(4):e0176139. doi: 10.1371/journal.pone.0176139 (PMC5405959; doi:10.1371/journal.pone.0176139)

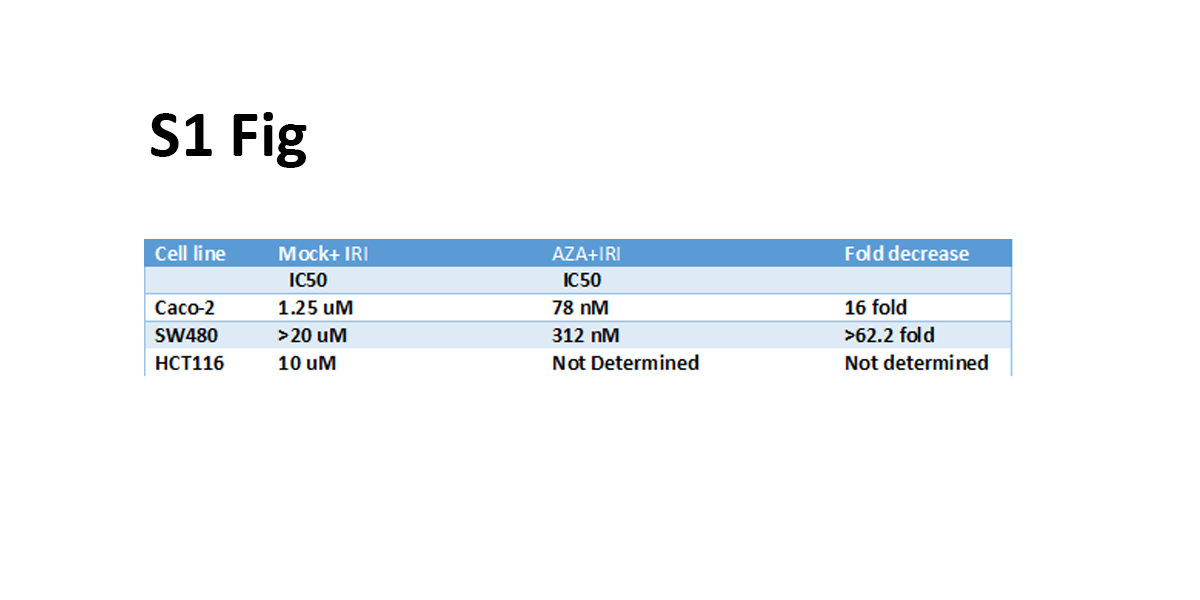

Supplement: S1 Fig — Three cell lines Caco-2, SW480, and HCT116 IC50 was determined. (TIF) [file pone.0176139.s001.tif]

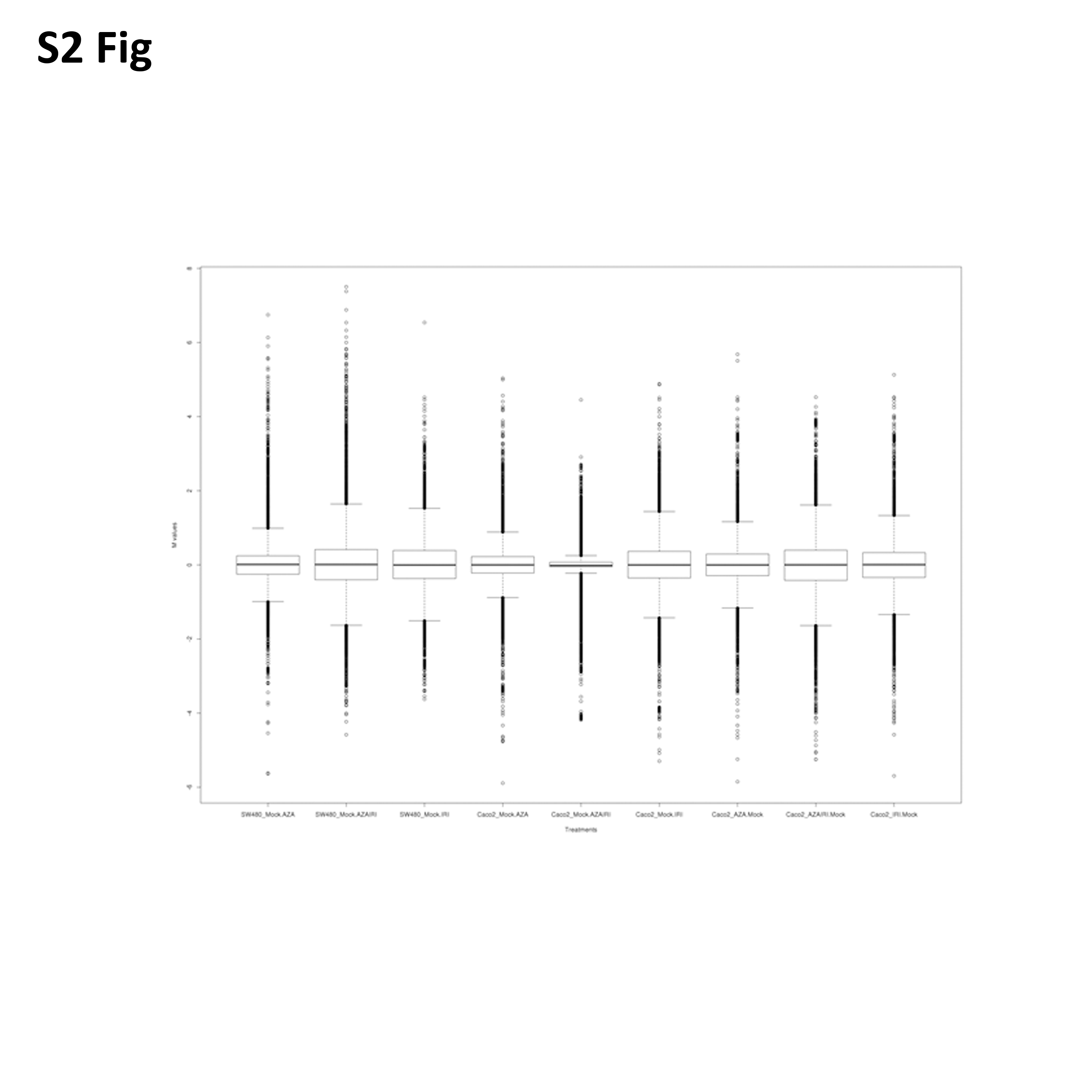

Supplement: S2 Fig — A log2 ratios between red and green signals in all nine arrays from this analysis following full normalization procedure (see Materials and methods for details). (TIF) [file pone.0176139.s002.tif]

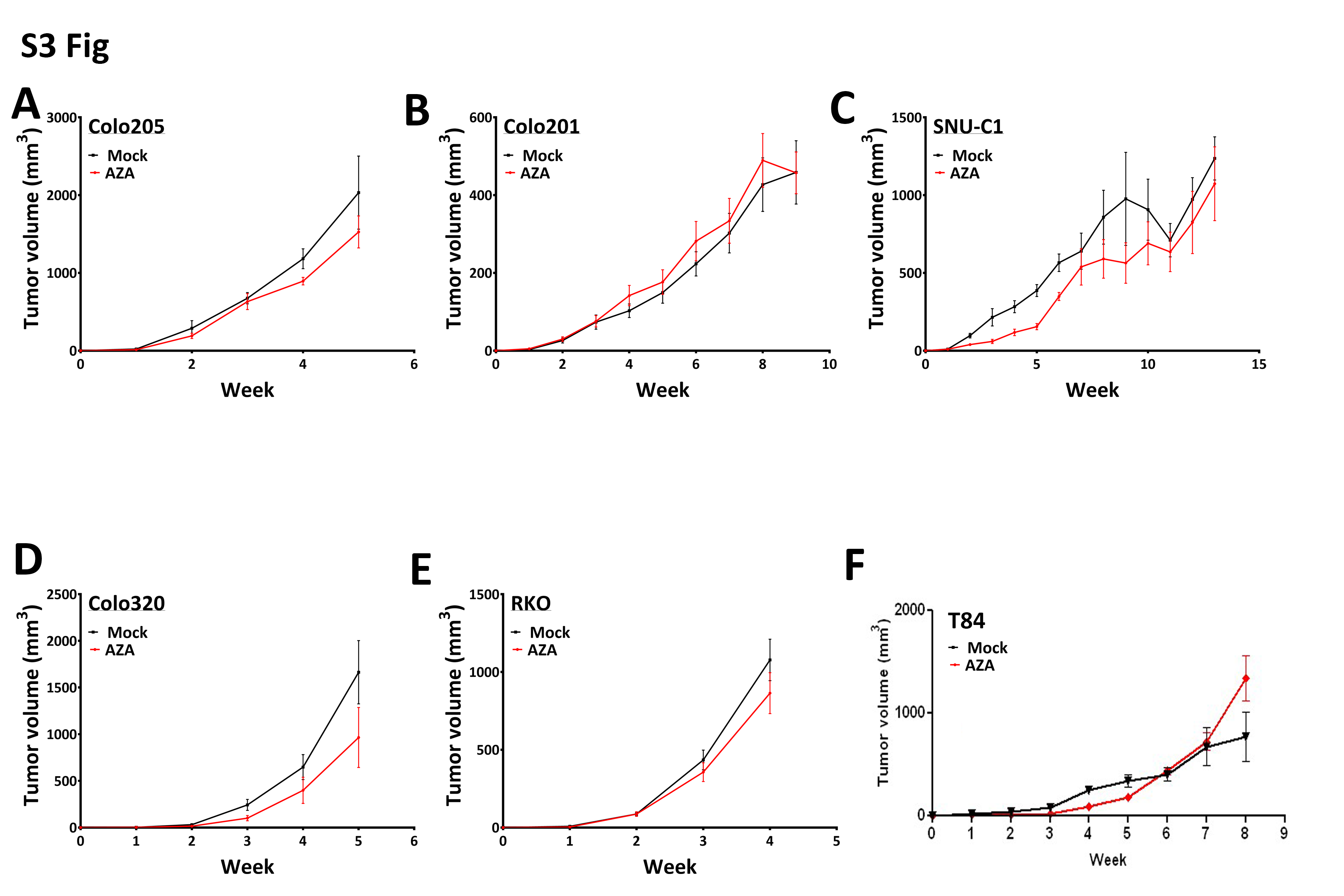

Supplement: S3 Fig — (A-F) Represents non-responder xenografted mice to the treatment. Mean tumor volume (±SEM) over time are plotted. Statistical significance determined by two-tailed paired t-test. (TIF) [file pone.0176139.s003.tif]
